# Environmental Risk Factors Associated With the Need for Penetrating Keratoplasty in Patients With Keratoconus

**DOI:** 10.7759/cureus.16506

**Published:** 2021-07-20

**Authors:** Khaled A Elubous, Muawyah Al Bdour, Taher Alshammari, Issa Jeris, Saif Aldeen AlRyalat, Allaa Roto, Mohammed Abu Ameerha

**Affiliations:** 1 Department of Ophthalmology, The University of Jordan, Amman, JOR

**Keywords:** consanguinity, counseling, jordan, keratoconus, middle-east, penetrating keratoplasty, risk factors

## Abstract

Purpose

To identify environmental risk factors associated with the need for penetrating keratoplasty (PKP) (full-thickness corneal transplantation) in patients with keratoconus in a Middle Eastern country.

Methods

This is a retrospective case-control study. This study included patients with keratoconus who underwent PKPor were waitlisted for PKP. Controls were patients diagnosed with keratoconus who did not reach a stage that necessitates PKP. Groups were matched by age and gender. Chi-square test was used to figure out the association between different risk factors including eye rubbing, vernal keratoconjunctivitis (VKC), smoking, paternal consanguinity, eye dryness, family history, asthma, eczema, and diabetes with the need for PKP.

Results

A total of 111 patients were included in this study, there were 48 (43.26%) men and 63 (56.75%) women. The case group included 42 subjects and the control group included 69 subjects. We found statistically significant differences between the two groups in relation to eye rubbing (p=0.0005), VKC (p=0.005), paternal consanguinity (p=0.02), and smoking rate (p=0.04), all being significant in the group in need of PKP. On the other hand, we did not find out a statistically significant difference between the two groups in relation to family history (p=0.31), dryness (p=0.58), asthma (p=0.15), eczema (p=0.28), or diabetes (p=0.29).

Conclusion

This study has identified several risk factors associated with the need for PKP in patients with keratoconus, part of which are modifiable. These findings can benefit clinicians in community counseling and give recommendations that can help in preventing or - at least - delaying the need for PKP surgery in keratoconus, such as smoking cessation, aggressive treatment of VKC disease, eye rubbing avoidance, as well as raising awareness regarding the potential risks of paternal consanguinity in this disease entity.

## Introduction

Keratoconus is a common bilateral ectatic corneal disease in the Middle-East region. It typically presents in young adults between the second and fourth decade of life [1]. The prevalence of keratoconus in Jordan is not yet determined but it is estimated to affect 1.38 people per 1,000 worldwide populations [2]. It is characterized by progressive corneal thinning and steepening [3]. Several environmental and genetic risk factors for keratoconus have been identified [4,5]. These risk factors are not limited to eye rubbing, dry eye, male sex, and screen time [6,7]. Geographical variation of sun exposure time and ethnicity are other risk factors that may explain the higher prevalence of this disorder in the Middle East countries [1]. Keratoconus can affect the vision in variable degrees ranging from mild to severe; however, most of the affected patients are in the mild form at the initial assessment [8]. Diagnosis of keratoconus requires assessment of visual acuity, slit-lamp examination, refraction, and the corneal topographic parameters [9]. Classification of keratoconus used to be determined using the Amsler-Krumeichand is replaced by a new classification system (ABCD), which takes into consideration the anterior and posterior radius of curvature at the central 3 mm zone as well as the corneal pachymetry and the distance best-corrected visual acuity [10]. Regarding treatment plan, this depends on many factors including the presence of corneal scarring, visual acuity, corneal thickness as well as the presence of ectasia progression. Based on these factors, the treatment option may be spectacles correction or contact lenses, intrastromal corneal ring segments, corneal cross-linking, or keratoplasty surgery [11]. Keratoplasty is also called “corneal transplantation,” where the diseased cornea is replaced by a healthy one from a human donor. It can be penetrating (full-thickness), deep anterior lamellar, or Bowman layer transplantation [12].

The aim of this study is to identify environmental risk factors associated with the need for penetrating keratoplasty (PKP) in patients with keratoconus in a Middle Eastern country, Jordan.

## Materials and methods

Patients

This study was conducted at the Jordan University Hospital. Institutional Review Board approval was obtained from Jordan University Hospital's ethical committee. The medical records for keratoconus patients who visited the clinics between January 2015 and December 2018 were reviewed. We confirmed the diagnosis by reviewing the patient's Pentacam (Oculus, Germany), refraction, and clinical data. We used a data sheet that contained demographic variables, including age and gender, environmental risk factors for keratoconus including the history of eye rubbing, vernal keratoconjunctivitis (VKC), smoking, asthma, eczema, eye dryness, diabetes mellitus, consanguinity between parents, family history of keratoconus (first and second degree), previous history of keratoplasty surgery or being on the waiting list for PKP. Patients were contacted by phone or face to face at the clinics (if available) to take their consent to participate in the study and fill the datasheet of the risk factors. We excluded patients with missing clinical data, having other ocular disorders, or patients who were uncertain about the degree of relativity between their parents or family history. We did not include patients if there is another family member previously included in the study to avoid overestimation of the risk of paternal consanguinity or the family history. In cases of bilateral keratoconus, only the more affected eye with lower visual acuity was included.

Statistical analysis

We used SPSS version 21.0 (IBM SPSS Statistics, Chicago, USA) in our analysis. The mean (± standard deviation) was used to describe continuous variables (i.e., age) and count (frequency) to describe other nominal variables (i.e., gender). One-Way ANOVA test was applied to analyze the mean difference in age between the two groups. We used the Chi-square test to analyze the difference between the two groups with each variable. All underlying assumptions were met unless otherwise indicated. We adopted a p-value of 0.05 as a significant threshold.

## Results

A total of 111 patients were included in this study, with a mean age of 31.21 (±12.47) years. They were 48 (43.25%) men and 63 (56.75%) women. The case group (who had PKP or were on the waiting list for PKP) had 42 subjects and the control group (not in need for PKP) had 69 subjects. The difference between the two groups in mean age and gender is not significant (p-value=0.43) and (p-value =0.26), respectively. Table 1 details the characteristics of patients included in this study.

**Table 1:** Demographics and risk factors for all the patients included in the study. SD - standard deviation; LogMAR - logarithm of the minimum angle of resolution

Variables	Cases	Control	Total	P-value
Male	21	27	48 (43.24%)	-
Female	21	42	63 (56.75%)	-
Gender%	-	-	-	0.262
Mean age of subjects±SD	32.40±11.32	30.49±13.15	31.21±12.47	0.436
Mean LogMAR visual acuity±SD	0.63±0.55	0.30±0.27	0.427±0.43	0.000068
Mean spherical equivalent refraction±SD	-2.46±3.40	-3.67±3.61	-3.21±3.57	0.083
Eye rubbing	35 (83.3%)	35 (50.7%)	73 (63.06%)	0.0005
Vernal keratoconjunctivitis	13 (30.95%)	7 (10.1%)	20 (18.01%)	0.005
Dryness	18 (42.85%)	26 (37.68%)	44 (39.63%)	0.588
Family history	23 (54.76%)	31 (44.92%)	54 (48.64%)	0.314
Paternal consanguinity	18 (42.86%)	16 (23.18%)	34 (30.63%)	0.029
Smoking	16 (38.09%)	14 (20.28%)	30 (27.02%)	0.040
Asthma	2 (4.76%)	9 (13.04%)	11 (9.90%)	0.156
Eczema	0	3 (4.34%)	3 (2.70%)	0.288
Diabetes mellitus	3 (7.14%)	2 (2.89%)	5 (4.50%)	0.295

We found a significant difference in eye rubbing (p=0.0005), with 35 (83.3%) of cases reported the previous history of eye rubbing compared to 35 (50.7%) for controls. A significant difference in VKC was also found (p=0.005), with 13 (30.95%) of cases reported the previous history of VKC compared to 7 (10.1%) controls. We found a significant difference in paternal consanguinity (p=0.029) and smoking rate (p=0.04) between the two groups. We did not find a significant difference between the two groups in terms of family history (p=0.314), dryness (p=0.588), asthma (p=0.156), eczema (p=0.288), and diabetes mellitus (p=0.29). The mean logarithm of the minimum angle of resolution (LogMAR) visual acuity for the subjects in cases was 0.63±0.55. For the controls, the mean LogMAR visual acuity was 0.30±0.27. The difference in visual acuity is statically significant with p-value = 0.000068. The mean spherical equivalent refraction for the cases was -2.46±3.40 and for the controls was -3.67±3.61, which is statically insignificant (p-value = 0.083).

## Discussion

To identify the risk factors associated with the need for PKP in patients with keratoconus disease, we compared two groups of patients with keratoconus. The case group consists of patients with keratoconus who underwent PKP surgery or being on the waiting list for PKP, while the control was age and gender-matched consisting of keratoconus patients who did not reach a stage that necessitates PKP.

In this study, we found a statistically significant association between the history of VKC and the need for PKP (p=0.005). This finding is consistent with previous studies that found out a strong association between VKC and severe keratoconus. Cingu et al. suggested a special assessment of patients presented with a history of VKC, as they are more prone to develop severe keratoconus at an earlier age and associated with high intraocular pressure [13,14]. VKC is also more common in Middle-East countries, which may contribute to the high incidence of keratoconus in these countries [15].

We found a statistically significant association (p=0.02) between parental consanguinity and the need for PKP. Consanguinity is common in many Middle Eastern countries [16]. About one-third of all marriages in Jordan were consanguineous marriages, and most were first cousin marriages [17]. In agreement with the population study, one-third of all patients reported a history of paternal consanguinity. The association of keratoconus and parental consanguinity has been previously reported [18-20]. However, its association with severe keratoconus or the need for PKP has not been previously evaluated. This point should be raised during history taking and assessment of patients with keratoconus. The association between consanguinity and the need for PKP may suggest an autosomal recessive inheritance pattern that may be associated with the severe presentation of keratoconus.

Similar to what Szczotka-Flynn et al. have reported; no significant association between family history and the need for PKP was found in our analysis [21]. Gaskin et al. reported that lack of family history was associated with acute corneal hydrops [22]. On the contrary, Naderan et al. found a significant association between family history and the severity of keratoconus [23]. The genetic background of keratoconus is not entirely understood, and further research is needed, especially in family-based studies and functional genomic approaches including epigenetics.

In agreement with previous studies, we found a statistically significant association between eye rubbing (p=0.0005) and the need for PKP [14,19]. About 60% of total subjects reported a history of eye rubbing, a similar observation was seen by Shneor et al. [24]. Moreover, eye rubbing can induce ocular surface inflammation, corneal thinning, and further progression of keratoconus [18].

 Smoking is not considered a risk factor for the development of keratoconus and may be negatively associated with it [25,26]. The association of smoking with the severity of keratoconus has been studied by Sahebjada et al. They did not find a significant association between smoking and the severity of keratoconus. However, we found a significant association between smoking and the need for PKP [27].

In our analysis, asthma, eczema, and diabetes were not found to be statistically associated with the need for PKP. Similarly, Sahebjada et al. did not find a significant association between these variables and the severity of keratoconus. On the other hand, Naderan et al. found a negative association between diabetes and severe forms of keratoconus [23,27].

Identifying the risk factors associated with the need for PKP is important in the management of patients with keratoconus. Several studies have reported these factors but a limited number of studies were conducted in an Asian country despite the higher prevalence of the disorder in these countries [8,28,29]. Up to our knowledge, this is the first study that evaluates the association between paternal consanguinity and the need for PKP.

Limitations

This study did not address the associations between keratometric parameters and corneal thicknesses with the need for PKP. However, our aim in this study was to evaluate the association between environmental risk factors and the need for PKP. Moreover, despite the importance of the sun exposure factor, we did not study it due to difficulty to determine the degree of sun exposure and the similarity of exposure across the geographical parts of the country.

## Conclusions

Our study has identified several environmental risk factors associated with the need for PKP surgery in patients with keratoconus, part of which are potentially modifiable. These results can benefit clinicians in patient and community counseling. Moreover, they help clinicians assess factors that are associated with a poor prognosis in those patients and give recommendations that help to control the modifiable risk factors to prevent or - at least -delay the need for PKP surgery, such as smoking cessation, aggressive treatment of VKC disease, eye rubbing avoidance as well as raising awareness regarding the potential risks of paternal consanguinity in this disease entity.
